# Death of Sir Benjamin C. Brodie

**Published:** 1862-12

**Authors:** 


DEATH OF SIR BENJAMIN C. BRODIE.—We regret to announce the death of Benjamin Collins Brodie, one of the most distinguished names in the annals of British surgery. He was born at Winterboro', in 1783, educated in a London free school and at St. George's Hospital, where he became the successor of Sir Everard Home as surgeon. In 1811 he received for his admirable physiological papers the Copley medal of the Royal Society. In 1819 he was appointed Professor of Anatomy in the Royal College of Surgeons, and in 1827, on the death of Sir Astley Cooper, became surgeon to the royal family, and attended King George IV. in his last illness. In 1850 he received the Degree of D. C. L. from Oxford. His baronetcy, bestowed upon him by William IV., dates from 1834. On the accession of Queen Victoria to the throne he was retained as "Sergeant Surgeon" to the royal family, and was till his death, October 21st, a personal friend of the Queen's. His last official appointment was the Presidency of the Royal Society, to which he was elevated in 1858. He was married in 1818, and leaves a widow and two sons—Benjamin Collins Brodie, Professor of Chemistry in the University of Oxford, and Rev. William Brodie, a clergyman of the Established Church.—*Boston Medical and Surgical Journal.*

